# Custom-made ocular prosthesis: solitary method to improve facial aesthetics (a case report)

**DOI:** 10.11604/pamj.2023.46.86.29680

**Published:** 2023-11-20

**Authors:** Neha Hajira, Atulya Sharma, Krutika Dang, Hebbal Shadaksharappa Shashidhara, Pulkit Khandelwal

**Affiliations:** 1 Department of Prosthodontics, Rural Dental College, Pravara Institute of Medical Sciences University Deemed to be University (PIMS-DU), Loni, Ahmednagar, Maharashtra, India,; 2 Department of Prosthodontics, College of Dental Sciences, Davangere, Karnataka, India,; 3 Department of Oral and Maxillofacial Surgery, Rural Dental College, Pravara Institute of Medical Sciences University (PIMS-DU), Loni, Ahmednagar, Maharashtra, India

**Keywords:** Aesthetics, eye, maxillofacial, ocular, case report

## Abstract

Loss of facial structures has a physical, social and psychological impact on affected individuals. Ocular trauma caused by assaults, falls, road traffic accidents, and household or work-related accidents may necessitate eye enucleation. Enucleation is also performed to treat severe infections of the eye, intraocular tumours and painful blind eyes. This procedure can result in post-enucleation socket syndrome with facial disfigurement and severely compromising facial aesthetics. Our patient complained of facial disfigurement because of a defect associated with the right eye. History and clinical features were suggestive of post-enucleation socket syndrome. The defect was rehabilitated with an ocular prosthesis. The rehabilitation procedure provided satisfactory results and a happy patient with good aesthetics. Maxillofacial prosthesis can restore and rehabilitate lost facial structures with artificial substitutes and helps in regaining patient´s natural appearance, the health of adjoining structures and subsequently, provide physical, social and psychological well-being.

## Introduction

Distress and suffering over the loss of any part of the body such as a limb, eye, nose, ear or finger have inimical and detrimental effects on behaviour, psychology and social well-being of patient. The eyes play a vital role as being a basic and important sense of sight. From vision to facial expression, eyes have numerous functions. Damage or loss of the eye critically hampers not only vision but also self-confidence. Absence or unfortunate loss of an eye may be caused due to any congenital anomaly, irreparable trauma or surgical intervention [1]. Ocular trauma occurs from motor vehicular accidents, assaults, falls, work-related trauma or injury from any sharp objects like broken glass, knife, screwdriver, nails, scissors, pencils or needles and may necessitate eye enucleation [2]. Enucleation is also indicated for the treatment of severe life-threatening infections of the eye, intraocular tumors and painful blind eyes [3,4]. Following enucleation of the eye, a decrease in orbital volume and displacement of intra-orbital structures lead to superior sulcus deepening, ptosis, ectropion, enophthalmos and laxity of eyelids, which is collectively termed as post-enucleation socket syndrome [5]. To restore and rehabilitate these ocular defects in post-enucleation socket syndrome, the only non-invasive treatment option available is ocular prosthesis.

This clinical report describes the management of post-enucleation socket syndrome to achieve marked improvement in facial esthetics by rehabilitation with ocular prosthesis in a young female patient. The ocular prosthesis corrected the ptosis, superior sulcus deepening, as well as enophthalmos.

## Patient and observation

**Patient information:** a 25-year-old female reported to our department with chief complaint of facial disfigurement because of a defect associated with her right eye. Her right eye was enucleated at age of 12 years following accidental injury with pencil at home. Medical history was non-significant.

**Figure 1:**
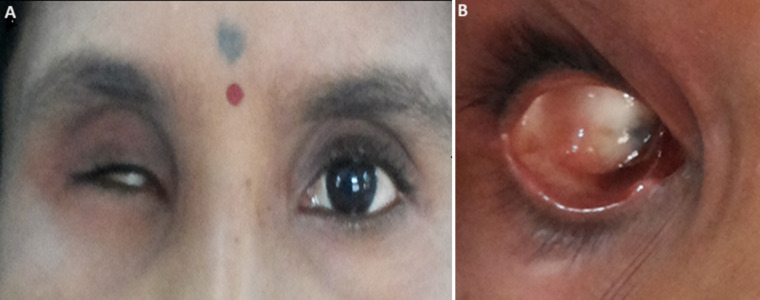
A) facial appearance of a patient with right eye defect with shrunken, superior sulcus deepening, ptosis of upper eyelid, and laxity of superior and inferior eyelids; B) and tissue bed of the ocular defect

**Clinical findings:** local clinical examination revealed a shrunken orbit having intact muscle bed with residual movements, superior sulcus deepening, ptosis of the upper eyelid and laxity of superior and inferior eyelids (Figure 1A). Conjunctiva was healthy with no sign of inflammation or infection. Palpebral fissure, palpebral muscle control and internal anatomy of the eye socket were evaluated. Sufficient depth and space between the conjunctival fornices was present to provide good retention to the prosthesis (Figure 1B). History and clinical features were suggestive of post-enucleation socket syndrome.

**Figure 2:**
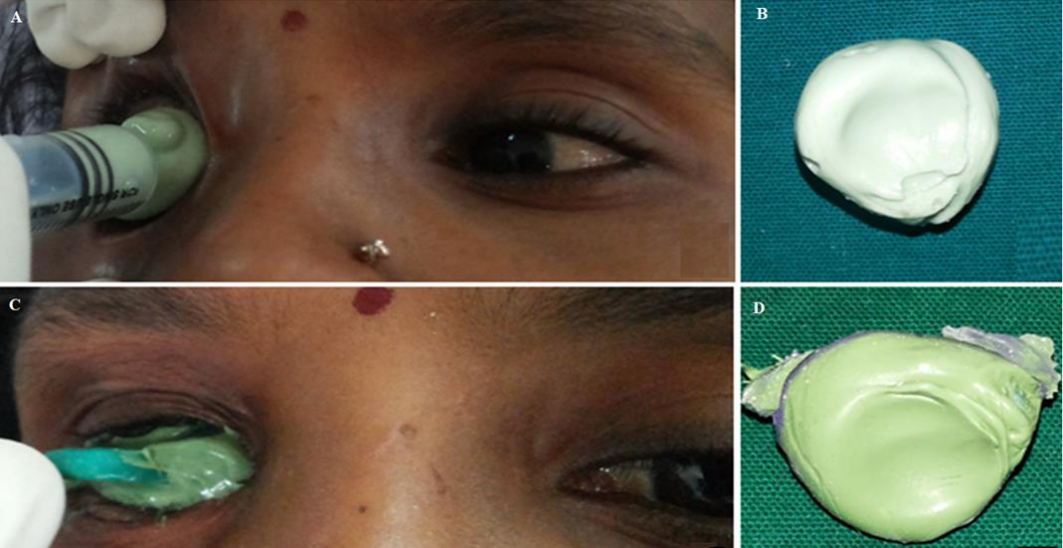
impression making procedure; A) primary impression injected with a syringe; B) primary impression of an ocular defect made with alginate; C) secondary impression made with custom tray; D) and secondary impression of ocular defect made with polyvinyl silicone impression material

**Therapeutic intervention:** treatment plan involved rehabilitation of ocular defect with custom-made ocular prosthesis. Procedure was explained to the patient and relatives, and informed consent was taken. Primary impression of right eye socket was taken with alginate (Figure 2 (A,B)) and a custom tray using auto-polymerized resin was fabricated over the primary stone cast. The final impression was made using light-bodied polyvinyl silicone impression material (Figure 2 (C,D)) and a stone cast was fabricated. The scleral wax pattern was fabricated using modelling wax and was tried in the ocular defect and assessed for contour, comfort, fit, aesthetics, support, size, simulation of eye movement, and eyelid coverage. Digital reproduction of the iris was done. This tried wax pattern was then processed in tooth-colored heat cure acrylic resin to form a scleral blank. Then the processed scleral blank was finished, polished and tried in socket defect. Eye contour, lid configurations and lid closure over scleral blank were re-evaluated to confirm symmetry with contralateral normal eye. The finished and polished eye prosthesis was disinfected using a solution of 70% isopropyl alcohol and 0.5% chlorhexidine. After disinfection, the prosthesis was rinsed using a sterile normal saline solution and lubricated with ophthalmic lubricant. Prosthesis was delivered and post-insertion instructions were given.

**Figure 3:**
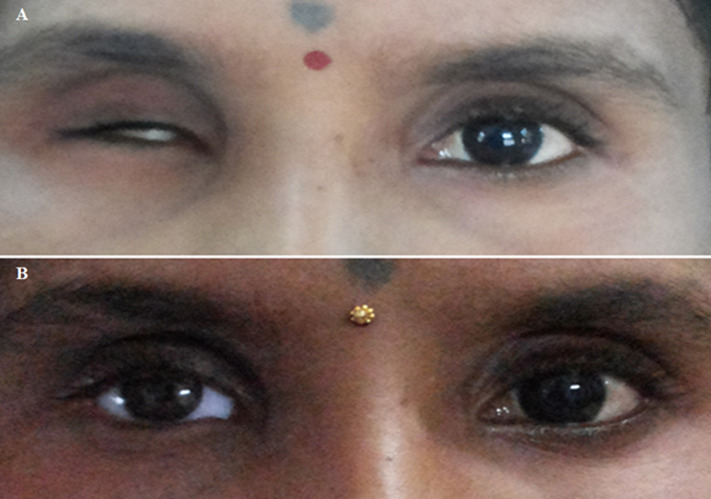
rehabilitation of post-enucleation socket; A) pre-operative photo of the ocular defect; B) and post-operative photo with a final ocular prosthesis

**Follow-up and outcomes and the patient’s perspective:** patient was followed up after one day, one week, one month and three months. The patient was satisfied with the outcome and there were no post-insertion complications like infection, loosening of prosthesis, etc. during regular follow-ups (Figure 3(A,B)).

**Informed consent:** written informed consent was obtained.

## Discussion

Post-enucleation socket syndrome causes marked facial disfigurement and can lead to significant physical, emotional and social trauma, mainly because of functional disability and societal reactions. Rehabilitation of a lost eye is imperative to aid in the physical and psychological healing of the patient and for better social appearance and acceptance. Ocular prosthesis is a maxillofacial prosthesis which restores the missing eye (eye bulb), maintains the volume of eye socket and simulates facial anatomy to re-create the impression of a perfectly normal healthy eye with normal surrounding tissues. It restores normal eye-opening, supports the eyelid, restores a degree of movement and appears aesthetically pleasing with reasonable motility [4-7]. The ocular prosthesis is a visual prosthetic; it does not provide vision. Patient donning an ocular prosthesis cannot see from the affected side and has monocular vision from a normal eye. Custom-made acrylic eye prosthesis is precise with good fit and superior aesthetics. Ocular prosthesis prevents collapse or loss of shape of eyelids, retains the shape of socket defect, provides adequate muscular function of eyelids, and prevents fluid accumulation in the ocular cavity subsequently enhancing tissue health and preventing bacterial growth. The prosthesis maintains a palpebral opening and gaze akin to the natural eye, and mimics the proportions and colouration of the natural eye. The prosthesis has close contact with tissue bed and its close adaptation favours even distribution of pressure reducing incidence of conjunctival injury (abrasion or ulceration). Exact colour and size matching of the iris, sclera and pupil with the opposite normal eye can be achieved. Even if the patient performs various movements, it maintains its orientation and position [6-9].

The goal of post-enucleation socket syndrome treatment is to achieve the best possible functional and esthetic result. The treatment can be either conservative or surgical. The conservative treatment is non-invasive and can be delivered with an ocular prosthesis. The volume deficit of the orbit can be corrected surgically with an alloplastic orbital implant [10]. In our case, the patient opted for a conservative manner of treatment and hence, an ocular prosthesis was fabricated. Properly and meticulously fabricated custom-made ocular prosthesis improved patient´s aesthetics, comfort and confidence, and maintained its orientation and position whenever the patient performed different eye movements.

## Conclusion

Rehabilitation of patients suffering from physical and psychological trauma of an eye loss needs a prudent prosthesis that will provide optimum aesthetic results and enhance physical and psychological healing with improved social appearance and acceptance.
